# Emotional and psychosexual well‐being is influenced by ethnicity and birthplace in women and individuals with polycystic ovary syndrome in the UK and India

**DOI:** 10.1111/1471-0528.17428

**Published:** 2023-03-08

**Authors:** Jameela Sheikh, Halimah Khalil, Salomi Shaikh, Meghnaa Hebbar, Nawal Zia, Saskia Wicks, Sindoora Jayaprakash, Alisha Narendran, Anuradhaa Subramanian, Kashish Malhotra, Rachel Chapman, Caroline Gillett, Helena K. Gleeson, Lynne Robinson, Justin J. Chu, Tejal Lathia, Chitra Selvan, Michael W. O'Reilly, Konstantinos N. Manolopoulos, Wiebke Arlt, Punith Kempegowda, the PCOS SEva team

**Affiliations:** ^1^ College of Medical and Dental Sciences University of Birmingham Birmingham UK; ^2^ DY Patil University School of Medicine Navi Mumbai India; ^3^ Barts Health NHS Trust London UK; ^4^ The Dudley Group NHS Foundation Trust Dudley UK; ^5^ King Edward VI High School for Girls Birmingham UK; ^6^ Institute of Applied Health Research University of Birmingham Birmingham UK; ^7^ Dayanand Medical College Ludhiana Punjab India; ^8^ University Hospitals Coventry and Warwickshire NHS Trust Coventry UK; ^9^ Institute of Metabolism and Systems Research University of Birmingham Birmingham UK; ^10^ Department of Endocrinology, Queen Elizabeth Hospital University Hospitals Birmingham NHS Foundation Trust Birmingham UK; ^11^ Birmingham Women's Hospital Birmingham Women's and Children's NHS Foundation Trust Birmingham UK; ^12^ Apollo Hospitals Navi Mumbai India; ^13^ Department of Endocrinology MS Ramaiah Medical College Bengaluru India; ^14^ Department of Medicine Royal College of Surgeons in Ireland (RCSI) University of Medicine and Health Sciences Dublin Ireland; ^15^ NIHR Birmingham Biomedical Research Centre at the University Hospitals Birmingham NHS Foundation Trust University of Birmingham Birmingham UK

**Keywords:** anxiety, body image, depression, obesity, polycystic ovarian syndrome, sexual function, weight stigma

## Abstract

**Objective:**

To assess the association of ethnicity and birthplace on emotional and psychosexual well‐being in women with polycystic ovary syndrome (PCOS).

**Design:**

Cross‐sectional study.

**Setting:**

Community recruitment via social media campaigns.

**Population:**

Women with PCOS completing an online questionnaire in September–October 2020 (UK) and May–June 2021 (India).

**Methods:**

The survey has five components, with a baseline information and sociodemographic section followed by four validated questionnaires: Hospital Anxiety and Depression Scale (HADS); Body Image Concern Inventory (BICI); Beliefs About Obese Persons Scale (BAOP); and Female Sexual Function Index (FSFI).

**Main outcome measures:**

We used adjusted linear and logistic regression models, adjusting for age, education, marital status and parity, to evaluate the impact of ethnicity and birthplace on questionnaire scores and outcomes (anxiety and/or depression, HADS ≥ 11; body dysmorphic disorder (BDD), BICI ≥ 72).

**Results:**

A total of 1008 women with PCOS were included. Women of non‐white ethnicity (613/1008) reported higher rates of depression (OR 1.96, 95% CI 1.41–2.73) and lower BDD (OR 0.57, 95% CI 0.41–0.79) than white women (395/1008). Women born in India (453/1008) had higher anxiety (OR 1.57, 95% CI 1.00–2.46) and depression (OR 2.20, 95% CI 1.52–3.18) but lower BDD rates (OR 0.42, 95% CI 0.29–0.61) than women born in the UK (437/1008). All sexual domains, excluding desire, scored lower for non‐white women and women born in India.

**Conclusions:**

Non‐white women and women born in India reported higher emotional and sexual dysfunction, whereas white women and women born in the UK reported higher body image concerns and weight stigma. Ethnicity and birthplace need to be considered for tailored, multidisciplinary care.

## 1 INTRODUCTION

Polycystic ovary syndrome (PCOS) is one of the most common endocrine disorders, affecting an estimated 10%–15% of women of reproductive age, with multi‐system manifestations.^1^ Several recent studies have shown PCOS is a lifelong condition with a significant risk of various metabolic disorders, rather than previously being thought of as a benign condition restricted to reproductive age.^2^, ^3^ The prevalence varies worldwide: in the UK, diagnosis ranges from 2.2% to 26% based on ethnic background; similarly, in India, PCOS is diagnosed in up to 22.5% of women of reproductive age in India.^4^, ^5^, ^6^, ^7^ Women with PCOS present with a range of reproductive, psychological or metabolic symptoms; however, women of South Asian origin disproportionately suffer more symptoms than any other ethnic group.^5^, ^8^, ^9^, ^10^ Current literature highlights ethnic differences in the clinical manifestation of PCOS, with the proposal that women of non‐white ethnicity require different PCOS diagnostic criteria and need a specialised tailored approach to tackling the complex genetic and environmental factors related to ethnicity, linked with obesity and associated metabolic conditions seen in different ethnic groups.^6^, ^7^, ^11^, ^12^, ^13^ However, these works have been limited to studies on women and individuals in high‐income countries. To the best of our knowledge, no studies have compared this difference across geographical borders, especially from low‐ and middle‐income countries. A systematic review of the lived experiences of women and individuals with PCOS also showed a need for tailored and culturally specific PCOS advice and management.^14^ Therefore, there is a need to understand the impact of ethnicity on PCOS. Country of birth has been used as an objective indicator for the identification of ethnic groups, avoiding self‐defining limitations of ethnic group classifications, and can be used as a proxy for country of origin and, therefore, comparison of different healthcare systems and cultures.^15^ The current literature investigating PCOS highlights the negative emotional impact, with a documented increased presence of depression and anxiety symptoms among women with PCOS.^4^, ^5^, ^16^

Body image is the mental perception of one's body and attitude towards one's physical self, appearance, health, sexuality and normal functioning.^17^ Persistent dissatisfaction with one's appearance, leading to chronic distress and worry that impacts day‐to‐day activities, is referred to as body dysmorphic disorder (BDD).^18^ BDD has been shown to adversely affect psychological functioning, increasing the risk of depression, anxiety and self‐harm among sufferers.^19^ However, few studies have evaluated perceived body image in women with PCOS and, by extension, the prevalence of BDD in women with PCOS.^17^

Female sexual dysfunction (FSD) is a persistent disorder causing difficulty in experiencing personally satisfying sexual activity.^20^ The prevalence of FSD is estimated at 21%–28% among premenopausal women.^21^ FSD is associated with obesity, metabolic syndrome and androgen excess, which are commonly seen in women with PCOS.^22^ Although some studies reveal a higher prevalence of FSD in women with PCOS, concerns persist around the methodological quality of the literature available.^23^

The UK National Institute of Health and Care Excellence guidelines recommend that healthcare professionals ask patients with PCOS about emotional well‐being and concerns regarding body image during consultations. However, the guidelines lack tailored advice and instead provide signposts to generalised eating disorders, which may contribute to the underestimation of emotional distress in PCOS sufferers.^24^

Some studies have highlighted the varied prevalence of mental health conditions. However, alongside body dysmorphia and sexual dysfunction, there is limited evidence focusing on emotional and psychosexual well‐being for women with PCOS in the community. In particular, few articles have analysed the impact of these factors on an ethnic minority group, either globally or beyond women in western culture.^17^

We report the results of a cross‐sectional study estimating the prevalence of anxiety, depression, body image concerns (BDD), weight stigma and sexual dysfunction in women with PCOS in the community, stratified by ethnicity and country of birth. We compare emotional and psychosexual ill‐being in women with PCOS of non‐white and white ethnicities and those born in the UK and India.

## 2 METHODS

We conducted a cross‐sectional study utilising an online survey distributed via a social media campaign. Women who self‐reported a PCOS diagnosis confirmed by a healthcare professional were included. All responses without a self‐reported PCOS diagnosis were excluded from the analysis. A similar method to invite patients to complete an anonymous online survey has been successfully employed elsewhere.^25^, ^26^, ^27^

The online survey was accessible to English‐speaking participants and was formatted to include relevant options for India and the UK, e.g. schooling levels. The survey consisted of three sections. The first section included details and rationale for the survey, general data protection regulation and consent. Those who consented were directed to the next section, which requested information on the sociodemographic details of the participant and details about their diagnosis of PCOS. This was followed by the third section, which included four validated questionnaires.

1. Anxiety and depression
  We used the Hospital Anxiety and Depression Scale (HADS) to examine the patients’ levels of anxiety and depression based on a 14‐item scale, scored out of 21. A score of 8–10 is defined as a borderline case and a score ≥11 is defined as a case of anxiety or depression.^28^
2. Body image
  The Body Image Concern Inventory (BICI) assesses self‐reported measures of dysmorphic appearance concerns. This validated questionnaire consists of 19 questions designed to evaluate opinions and concerns about appearance. Each item uses a Likert‐scale rating of the frequency of feeling or performing the described behaviour. The BICI ranges from 19 to 95, with a score of ≥72 suggesting BDD.^29^, ^30^
3. Weight‐related stigma
  We used the Beliefs About Obese Persons Scale (BAOP) to examine beliefs about the causes of obesity. It is an eight‐item questionnaire where a higher score suggests weight‐related bias.^31^
4. Female sexual function
  The Female Sexual Function Index (FSFI) was used to assess the domains of sexual function: desire, arousal, lubrication, orgasm, satisfaction and pain. The score reflects overall sexual function, with a lower FSFI score suggesting psychosexual dysfunction.^32^

### 2.1 Design and data collection

We collected data during two time periods, September–October 2020, for women based in the UK, and June–July 2021, for women in India. We started our sample population by launching a targeted social media campaign centred on PCOS Awareness Month in September, in collaboration with two national patient support groups, Verity and PCOS Vitality.^33^, ^34^ In June and July 2021 we collaborated with two patient support groups in India, PCOS Loqus and PCOS Club India.^35^, ^36^ All participants were recruited via social media posts supported by patient groups and provided a direct link to the first introductory part of the online survey, as detailed above. Upon accessing the survey, the participants were provided with the information on the study with an inbuilt electronic consent process. They were provided information that all entries were anonymous and that we would not be able to identify or remove data once submitted. Participants could only progress with the survey once they consented.

### 2.2 Statistical analysis

Statistical analysis was performed using STATA 14.2 (StataCorp LLC, College Station, TX, USA). All exposure measures were calculated in accordance with the guidance of the validated questionnaires in the literature: HADS anxiety score, HADS depression score, BICI score, BAOP score and FSFI score. We reported median and interquartile ranges (IQRs) for the diagnoses scores and frequency and percentages for all patients and for patients stratified by birthplace and ethnic status. For the analysis, we re‐coded primary exposure variables, such as ethnicity and country of birth, as dichotomous variables because of the low sample numbers in each individual category. Ethnicity was grouped into white ethnic women (white British and white Irish women) and non‐white ethnic women, which included the remaining ethnic groups. For the country‐of‐birth analysis, we only grouped women into those born in the UK and those born in India.

We used logistic regression models to obtain adjusted odds ratios and 95% confidence intervals for each diagnostic outcome variable (anxiety, depression and BDD), derived from the cut‐off scores. We used linear regression models to obtain adjusted beta coefficients (β) and 95% CIs for each continuous outcome variable (HADS anxiety and depression scores, BICI scores, BAOP scores and FSFI domain scores). We adjusted for age category, level of education, marital status and parity in the logistic and linear regression models.

## 3 RESULTS

### 3.1 Composition of the overall cohort and overall emotional and psychosexual well‐being

The survey received responses from 1060 women who consented, 1008 (95.1%) of whom had a self‐reported diagnosis of PCOS made by a healthcare professional and were included in the study. We received responses from women living in 30 countries: 50.7% (440/868) in India; 39.6% (344/868) in the UK; 2.8% (24/868) in the USA; 1.4% (12/868) in Australia; six women from the United Arab Emirates; five each from the Philippines and Ireland; four from Germany; two each from Canada, France, Malaysia, Nigeria, Pakistan and Sri Lanka; and one each from Afghanistan, Argentina, Austria, Barbados, Georgia, Iran, Japan, Kuwait, the Netherlands, Oman, Peru, Qatar, Singapore, South Africa, Sweden, and Trinidad and Tobago. We could not perform further analysis into ethnicity and country of birth based on country of residence because of limited subgroup numbers.

The majority of the participants were of non‐white ethnicity (60.8%, 613/1008). For country of birth, 44.9% (453/1008) of women were born in India and 43.4% (437/1008) were born in the UK. Most of the participants were in the age category of 26–35 years (44.8%), had an undergraduate degree (44.9%), were in full‐time employment (47.6%), were single (53.9%), and did not have children (84.1%). The full characteristics are presented in Table 1.

**TABLE 1:** Characteristics of the study population.

Variable	Frequency, *n* (%)				
	Overall	Ethnicity		Country of birth	
		Non‐white	White	India	UK
Total	1008	613	395	453	437
Age group (years)					
17 or younger	10 (1.0)	8 (1.3)	2 (0.5)	6 (1.3)	2 (0.5)
18–25	390 (38.7)	308 (50.2)	82 (20.8)	241 (53.2)	111 (25.4)
26–35	452 (44.8)	260 (42.4)	192 (48.6)	195 (43.0)	200 (45.8)
36–45	128 (12.7)	33 (5.4)	95 (24.1)	11 (2.4)	101 (23.1)
46–55	26 (2.6)	4 (0.7)	22 (5.6)	0 (0)	21 (4.8)
56 or older	2 (0.2)	0 (0)	2 (0.5)	0 (0)	2 (0.5)
Ethnicity					
Asian or Asian British Bangladeshi	5 (0.5)	5 (0.8)	0 (0)	0 (0)	4 (0.9)
Asian or Asian British Indian	487 (48.3)	487 (79.4)	0 (0)	452 (99.8)	14 (3.2)
Asian or Asian British Pakistani	10 (1)	10 (1.6)	0 (0)	0 (0)	7 (1.6)
Black or Black British African	8 (0.8)	8 (1.3)	0 (0)	0 (0)	1 (0.2)
Black or Black British Caribbean	11 (1.1)	11 (1.8)	0 (0)	0 (0)	9 (2.1)
Mixed Background	23 (2.3)	23 (2.3)	0 (0)	1 (0.2)	11 (2.5)
White British	384 (38.1)	0 (0)	384 (97.2)	0 (0)	371 (84.9)
White Irish	11 (1.1)	0 (0)	11 (2.8)	0 (0)	10 (2.3)
Other	69 (6.8)	69 (6.8)	0 (0)	0 (0)	10 (2.3)
Education					
Postgraduate degree	283 (28.1)	216 (35.2)	70 (17.7)	180 (39.7)	77 (17.6)
Undergraduate degree	453 (44.9)	306 (49.9)	147 (37.2)	232 (51.2)	166 (38.0)
A‐levels or equivalent	184 (18.3)	69 (11.3)	115 (29.1)	31 (6.8)	129 (29.5)
GCSE or equivalent	56 (5.6)	6 (1.0)	51 (12.9)	3 (0.7)	52 (11.9)
No formal qualifications	6 (0.6)	3 (0.5)	3 (0.8)	0 (0)	3 (0.7)
Other	26 (2.6)	13 (2.1)	9 (2.3)	7 (1.5)	10 (2.3)
Occupation					
Full‐time employment	480 (47.6)	243 (39.6)	237 (60.0)	156 (34.4)	255 (58.4)
Part‐time employment	158 (15.7)	79 (12.9)	79 (20.0)	50 (11.0)	88 (20.1)
Retired	2 (0.2)	0 (0)	2 (0.5)	0 (0)	2 (0.5)
No paid work	368 (36.5)	291 (47.5)	77 (19.5)	247 (54.5)	92 (21.1)
Marital status					
Married/civil partnership	342 (33.9)	179 (29.2)	163 (41.3)	135 (29.8)	166 (38.0)
Cohabitating	107 (10.6)	21 (3.4)	86 (21.8)	4 (0.9)	91 (20.8)
Separated/divorced	16 (1.6)	7 (1.1)	9 (2.3)	5 (1.1)	8 (1.8)
Single	543 (53.9)	406 (66.2)	137 (34.7)	309 (68.2)	172 (39.4)
Number of children					
0	848 (84.1)	573 (93.5)	275 (69.6)	431 (95.1)	317 (72.5)
1	84 (8.3)	23 (3.8)	61 (15.4)	17 (3.8)	59 (13.5)
2	60 (6.0)	12 (2.0)	48 (12.2)	5 (1.1)	49 (11.2)
3	15 (1.5)	5 (0.8)	10 (2.5)	0 (0)	12 (2.7)
4+	1 (0.1)	0 (0)	1 (0.3)	0 (0)	0 (0)

Investigating emotional well‐being, 60.6% (611/1003) and 24.3% (245/1003) had a diagnosis of anxiety (median 12, IQR 9–15) or depression (median 8, IQR 4–10), respectively (HADS ≥ 11). HADS scores indicative of both anxiety and depression were found in 8.3% (83/1003) of participants, whereas 13.8% (138/1003) of participants had neither anxiety nor depression. The prevalence of borderline diagnoses (HADS 8–10) of anxiety and depression, respectively, was 18.9% (191/1003) and 26.7% (269/1003) (Table S1).

Based on BICI scores, 38.5% (388/1005) of women were classified as having BDD (BICI ≥ 72); the median BICI score in the overall cohort was 66 (IQR 52–78). A total of 997 women completed the BAOP questionnaire and the median score was 30 (IQR 25–35), where a higher score indicates stronger beliefs that people with obesity cannot control their weight status.^37^ The overall results of the FSFI questionnaire had a median score of 20.2 (IQR 7.4–26.1); 73.9% of women who completed the FSFI questionnaire were sexually active (723/979) in the last 4 weeks. The median and IQR scores for the domains of the FSFI were as follows: desire (3.0, 2.4–4.2), arousal (3.0, 0.0–4.5), lubrication (3.9, 0.0–5.4), orgasm (3.2, 0.0–4.8), satisfaction (4.0, 2.8–4.8) and pain (2.0, 0.0–4.8). As described in the methods, a lower FSFI score suggests psychosexual dysfunction.^32^

### 3.2 Impact of ethnicity on emotional and psychosexual well‐being

Emotional, psychological and sexual well‐being were compared between women of non‐white ethnicity (613/1008) and women of white ethnicity (395/1008) (Table 2).

**TABLE 2:** Comparison of scores from validated questionnaires assessing the emotional, psychological and sexual well‐being of women with PCOS based on ethnicity, comparing non‐white ethnic women with white ethnic women.

Scores	Median (IQR)		Adjusted coefficient (95% CI)	*p*
	Non‐white ethnicity (*n* = 613/1008)	White ethnicity (*n* = 395/1008)		
Emotional well‐being				
HADS anxiety	12 (9–15)	11 (8–14)	0.523 (−0.158, 1.204)	0.132
HADS depression	8 (5–11)	7 (4–10)	1.331 (0.677, 1.985)	**<0.001**
Psychological well‐being				
BICI	61 (47–76)	72 (60–81)	−7.480 (−10.211, −4.749)	**<0.001**
BAOP	29 (23–34)	32 (28–35)	−2.698 (−3.944, −1.451)	**<0.001**
Sexual well‐being				
FSFI overall	18.15 (6.8–24.7)	23.3 (11.0–28.1)	−3.052 (−4.587, −1.517)	**<0.001**
FSFI desire	3.6 (2.4–4.2)	2.4 (1.8–3.6)	0.140 (−0.083, 0.362)	0.219
FSFI arousal	2.7 (0.0–4.2)	3.3 (1.2–4.8)	−0.399 (−0.725, −0.072)	**0.017**
FSFI lubrication	3.6 (2.0–4.8)	4.5 (0.3–5.7)	−0.528 (−0.900, −0.156)	**0.005**
FSFI orgasm	2.8 (0.0–4.8)	4.0 (0.0–5.2)	−0.479 (−0.836, −0.121)	**0.009**
FSFI satisfaction	3.2 (2.0–4.8)	4.4 (3.2–5.2)	−0.893 (−1.128, −0.659)	**<0.001**
FSFI pain	0.0 (0.0–4.4)	4.0 (0.0–5.6)	−0.893 (−1.265, −0.522)	**<0.001**

*Note*: Bold value indicates statistical significant.

Abbreviations: BAOP, Beliefs About Obese Persons Scale; BICI, Body Image Concern Inventory; FSFI, Female Sexual Function Index; HADS, Hospital Anxiety and Depression Scale.

Women with PCOS of non‐white ethnicity had higher scores of depression (β 1.33; 95% CI 0.68, 1.99; *p* < 0.001) and reported a higher incidence of depression (OR 1.96; 95% CI 1.41, 2.73; *p* < 0.001) (Table 3), compared with ethnically white women. A similar pattern was seen for anxiety, although this lacked statistical significance (Table 3).

**TABLE 3:** The impact of ethnicity and country of birth on a diagnosis of anxiety, depression and BDD.

Ethnicity					
Diagnosis	Non‐white ethnicity (*n* = 611)	White ethnicity (*n* = 392)	Adjusted odds ratio	95% CI	*p*
	Events (%)	Events (%)			
Anxiety	390 (63.6)	221 (55.9)	1.323	0.884, 1.981	0.174
Depression	161 (26.3)	84 (21.3)	1.963	1.410, 2.733	**<0.001**
BDD	201 (32.8)	187 (47.3)	0.568	0.408, 0.791	**0.001**

Country of birth					
Diagnosis	India (*n* = 453)	UK (*n* = 433)	Odds ratio	95% CI	*p*
	Events (%)	Events (%)			
Anxiety	297 (65.6)	248 (56.8)	1.573	1.002, 2.458	**0.049**
Depression	123 (27.2)	96 (22.0)	2.199	1.521, 3.177	**<0.001**
BDD	129 (28.5)	210 (48.1)	0.420	0.290, 0.610	**<0.001**

*Note*: Bold value indicates statistical significant.

Abbreviation: BDD, body dysmorphic disorder.

There were significant differences associated with ethnicity and psychosexual well‐being. Women of non‐white ethnicity had lower BICI scores (β −7.48, 95% CI −10.21, −4.75; *p* < 0.001) and BAOP scores (β −2.70; 95% CI −3.94, −1.45; *p* < 0.001), mirroring a lower prevalence of BDD in non‐white ethnic women compared with white ethnic women (OR 0.57; 95% CI 0.41–0.79; *p* = 0.001) (Table 3).

Women of white ethnicity had higher FSFI scores (β −3.05; 95% CI −4.59, −1.52; *p* < 0.001), denoting higher sexual well‐being compared with women of non‐white ethnicity. This significant difference translated to the majority of FSFI domains: arousal (β −0.40; 95% CI −0.73, −0.07; *p* = 0.017), lubrication (β −0.53; 95% CI −0.90, −0.16; *p* = 0.005), orgasm (β −0.48, 95% CI −0.84, −0.12; *p* = 0.009), satisfaction (β −0.89; 95% CI −1.13, −0.66; *p* < 0.001) and pain (β −0.89; 95% CI −1.27, −0.52; *p* < 0.001).

### 3.3 Impact of country of birth on emotional and psychosexual well‐being

Differences between emotional and psychosexual well‐being were investigated between participants born in the UK (437/1008) and India (453/1008) (Table 4).

**TABLE 4:** Comparison of scores of validated questionnaires assessing the emotional, psychological and sexual well‐being of women with PCOS based on country of birth, comparing women born in India with women born in the UK.

	Median (IQR)		Adjusted coefficient (95% CI)	*p*
	India (*n* = 453/1008)	UK (*n* = 437/1008)		
Emotional well‐being				
HADS anxiety	13 (9–15.5)	11 (8–14)	1.090 (0.341, 1.840)	**0.004**
HADS depression	8 (5–11)	7 (4–10)	1.637 (0.916, 2.359)	**<0.001**
Psychological well‐being				
BICI	58 (44–73.5)	72 (60–81)	−11.960 (−14.959, −8.960)	**<0.001**
BAOP	29 (23–34)	32 (27–35)	−3.384 (−4.768, −2.000)	**<0.001**
Sexual well‐being				
FSFI overall	17.1 (6.4–24.5)	22.45 (9.65–27.98)	−3.502 (−5.197, −1.807)	**<0.001**
FSFI desire	3.6 (2.4–4.2)	3.0 (1.8–3.6)	0.150 (−0.096, 0.397)	0.231
FSFI arousal	2.7 (0.0–4.2)	3.3 (1.2–4.8)	−0.431 (−0.791, −0.070)	**0.019**
FSFI lubrication	3.3 (0.0–4.8)	4.35 (0.3–5.7)	−0.598 (−1.010, −0.187)	**0.004**
FSFI orgasm	2.8 (0.0–4.8)	4.0 (0.0–5.2)	−0.595 (−0.988, −0.202)	**0.003**
FSFI satisfaction	2.8 (1.6–4.4)	4.4 (3.2–4.8)	−1.053 (−1.313, −0.794)	**<0.001**
FSFI pain	0.0 (0.0–4.0)	3.6 (0.0–5.6)	−0.975 (−1.381, −0.568)	**<0.001**

*Note*: Bold value indicates statistical significant.

Abbreviations: BAOP, Beliefs About Obese Persons Scale; BICI, Body Image Concern Inventory; FSFI, Female Sexual Function Index; HADS, Hospital Anxiety and Depression Scale.

Women with PCOS in India had lower emotional well‐being when compared with those born in the UK. The participants born in India reported a higher anxiety score (β 1.09; 95% CI 0.34, 1.84; *p* = 0.004) and higher prevalence of anxiety (OR 1.57; 95% CI 1.00, 2.46; *p* = 0.049) after adjusting for age, educational status, marital status and parity (Table 3). Similarly, women born in India reported higher depression scores (β 1.64; 95% CI 0.92, 2.36; *p* < 0.001) and a higher prevalence of depression (OR 2.20; 95% 1.52, 3.18; *p* < 0.001) (Table 3).

Women in the UK reported higher degrees of psychological ill‐being: higher BICI scores (β −11.96; 95% CI −14.96, −8.96; *p* < 0.001), higher prevalence of BDD (OR 0.42; 95% CI 0.29, 0.61; *p* < 0.001; Table 3) and BAOP scores indicative of weight stigma (β −3.38; 95% CI −4.77, −2.00; *p* < 0.001).

Participants born in the UK had higher FSFI scores overall (β −3.50; 95% CI −5.20, −1.81; *p* < 0.001), denoting improved sexual well‐being compared with participants born in India. This significance was reflected within the majority of FSFI domains: arousal (β −0.43; 95% CI −0.79, −0.07; *p* = 0.019), lubrication (β −0.60; 95% CI −1.01, −0.19; *p* = 0.004), orgasm (β −0.60; 95% CI −0.99, −0.20; *p* = 0.003), satisfaction (β −1.05; 95% CI −1.31, −0.79; *p* < 0.001) and pain (β −0.98; 95% CI −1.38, −0.57; *p* < 0.001).

## 4 DISCUSSION

### 4.1 Main findings

Our study found a significant association between ethnicity and birthplace on emotional and psychosexual well‐being in women with PCOS in the community. Our results support previous reports on the high incidence of anxiety, depression and body image concerns among women with PCOS.^38^ We show that women of white ethnicity and women born in the UK showed significantly higher body image concerns, weight stigma scores and prevalence of BDD, compared with women born in India and of non‐white ethnicity. Features of PCOS such as acne, hair loss, weight gain and hirsutism have been linked to lower levels of self‐esteem and greater fear of negative evaluation of appearance.^17^, ^39^ However, an ethnicity‐specific analysis of the impact of these features on emotional, psychological and sexual well‐being has not yet been carried out.

### 4.2 Strengths and limitations

Strengths of our study include the large sample size with equally sized participant cohorts from the UK and India and of white and non‐white ethnic origin. To the best of our knowledge, this is the first study investigating the impact of psychological well‐being, body image concerns and weight stigma in PCOS, further highlighting the importance of these results in revealing that country of birth and ethnicity may impact the phenotypic presentation of PCOS.^40^

A weakness of our study is the potential bias in recruitment, as this was primarily achieved through patient support groups via social media and, therefore, may have resulted in selection bias for women with a higher level of symptoms, as well as the inclusion of self‐reported diagnoses of PCOS. The survey was accessible to English‐speaking participants only, which may have prevented the inclusion of women not proficient in English. The cross‐sectional study design reduces the ability to minimise confounding factors, such as data collection during the coronavirus disease 2019 (COVID‐19) pandemic, which may have influenced well‐being. We collected the Indian cohort data during the peak of the COVID‐19 pandemic in India, replicating the conditions as best as possible to allow comparison between Indian and UK cohorts. Furthermore, the limited heterogeneity of ethnicity in country of birth group, e.g. non‐white ethnicity born in the UK compared with non‐white ethnicity born in India, and variation in country of birth within ethnicity groups, prevented secondary subgroup analysis. Overall, this study aimed mainly to highlight the problem, and further in‐depth analysis in the form of case–control methodology will consolidate these results and confirm the findings. In addition, results were adjusted for participant demographics, thereby minimising the influence of these factors and strengthening the conclusions. The analysis included a small proportion of women self‐classified as postmenopausal; however, this cohort was small and was therefore included as a representation of the community and is unlikely to change the trend. In this study, the absence of baseline controls not suffering from PCOS prevents us from confirming notable trends that can be attributed to PCOS alone, and confounding factors such as social and educational factors should be considered. All participants self‐reported a PCOS diagnosis by a healthcare professional, which could not be verified and may bias the results. Also, the cohort completing the survey may bias against those unable to access social media or online surveys. However, unlike other studies focusing on clinic attendance, this study aims to capture women in the community who are often neglected, and who may be more representative of the population with PCOS. We also acknowledge that the findings are not generalisable across all ethnicities, and future studies of women from various ethnic minorities are needed to dissect the difference in the impact of PCOS on emotional well‐being.

### 4.3 Interpretation

Investigation into the impact of anxiety and depression in women with PCOS is extensive. Barry et al. conducted a systematic review and meta‐analysis demonstrating that women with PCOS tend to experience mildly elevated levels of anxiety and depression,^41^ which supports the findings of a high prevalence of anxiety and depression in our cohort. Supporting our results, further analysis into the healthcare‐related costs of mental health disorders in women with PCOS revealed that these women were significantly more likely to have anxiety, depression and eating disorders, compared with women without PCOS.^42^ This may suggest an underestimation of the prevalence of emotional distress in the community, as the majority of studies focus on women attending secondary care services.

Sexual dysfunction in women with PCOS is important to explore, considering the potential impact of PCOS‐related clinical features, such as obesity, hyperandrogenism and anovulation. Eftekhar et al. investigated sexual dysfunction with a similar methodology, using the FSFI questionnaire, and concluded that women with PCOS suffered considerably from sexual dysfunction.^22^ A particular focus is required for women of non‐white ethnicity with potential sexual satisfaction dysfunction, which is also reflected in the population born in India. In addition, considering the cultural differences of openness when discussing domains of sexual function is important when interpreting FSFI results from different nationalities. This is supported by the literature, where it is noted that Indian women were less likely to discuss and seek assistance with sexual health with a healthcare professional, with socio‐economic factors and family tradition influencing disclosure.^43^ Our study confirms the need for appropriate screening of women with PCOS for sexual well‐being and proposes targeted interventions to bridge the health communication gaps in sexual health.

Future investigations on the impact of ethnicity are needed to inform guidelines to better tailor care for these women with a higher risk of suffering from psychological ill‐being. Further to this, a consideration of the impact of the environment is important to highlight, particularly where women born in the UK reported higher psychosexual ill‐being, which may be associated with societal stigma.

## 5 CONCLUSION

Our study investigating emotional and psychosexual well‐being in women with PCOS reveals high rates of anxiety, depression and body dysmorphic disorder, poor body image, weight stigma and sexual dysfunction. In particular, ethnicity and birthplace can influence this emotional and psychosexual ill‐being. This warrants the need for regular in‐depth universal screening and emphasises the role of a psychologist in the PCOS multidisciplinary team to provide comprehensive care and efforts to improve awareness among primary healthcare professionals when managing women with PCOS in the community.

## AUTHOR CONTRIBUTIONS

PK and JS conceptualised the study; WA, KNM, and MWO'R contributed to the study design. JS, SS, HK, and MH planned and were involved in carrying out the study. JS and AS conducted the analyses. All authors contributed to the writing of the manuscript and approved the final version. PK, WA, and JS are the guarantors. All authors agree with the final version and agree to be accountable for the integrity of the data published.

## Funding information

This work has been supported by the Wellcome Trust (investigator grant WT209492/Z/17/Z, to WA) and the Health Research Board (Emerging Clinician Scientist Award ECSA‐FA‐2020‐001, to MWOR). WA receives support from the National Institute for Health and Care Research (NIHR) Birmingham Biomedical Research Centre at the University Hospitals Birmingham NHS Foundation Trust and the University of Birmingham (grant ref. no. BRC‐1215‐20009). The views expressed are those of the authors and not necessarily those of the NIHR UK or the Department of Health and Social Care UK.

## CONFLICT OF INTEREST STATEMENT

None declared. Completed disclosure of interests form available to view online as supporting information.

## ETHICS APPROVAL

This study has received approval from the Institutional Ethics Committee – Biomedical Research, Apollo Hospitals, Navi Mumbai (application reference: AHM‐ACD‐021/02–21) and HRA and Health and Care Research Wales (HCRW) approval (IRA project ID: 299243; REC reference: 22/PR/1178).

## Supporting information


Table S1.

Data S1.

## Data Availability

The data underlying this article will be shared on reasonable request to the corresponding author.
