# Supplementary material for: Emotional and psychosexual well‐being is influenced by ethnicity and birthplace in women and individuals with polycystic ovary syndrome in the UK and India
Source: BJOG. 2023 Mar 8;130(8):978–86. doi: 10.1111/1471-0528.17428 (PMC10952802; doi:10.1111/1471-0528.17428)
Supplement: Supplementary file 1 — Table S1. [file BJO-130-978-s002.docx]

**Table S1***: Scores of validated questionnaires for the full cohort.*

|  | **N** | **Median (IQR)** | **Diagnostic Threshold** |
| --- | --- | --- | --- |
| ***Emotional wellbeing*** | | | |
| HADS Anxiety score | 1003 | 12 (9-15) | Score 8-10 borderline case  Score ≥ 11 case |
| HADS Depression score | 1003 | 8 (4-10) |  |
| ***Psychological wellbeing*** | | | |
| BICI (body image concern) | 1005 | 66 (52-78) | Score ≥ 72 BDD |
| BAOP (weight-related stigma) | 997 | 30 (25-35) | Higher score suggests weight-related bias |
| ***Sexual wellbeing*** | | | |
| FSFI Desire | 979 | 3.0 (2.4-4.2) | Lower score suggests psychosexual dysfunction |
| FSFI Arousal | 979 | 3.0 (0.0-4.5) |  |
| FSFI Lubrication | 979 | 3.9 (0.0-5.4) |  |
| FSFI Orgasm | 979 | 3.2 (0.0-4.8) |  |
| FSFI Satisfaction | 979 | 4.0 (2.8-4.8) |  |
| FSFI Pain | 979 | 2.0 (0.0-4.8) |  |
| FSFI Overall | 979 | 20.2 (7.4-26.1) |  |
|  | **N** | **Frequency (%)** | **Diagnostic Threshold** |
| ***Emotional wellbeing*** | | | |
| Anxiety diagnosis | 1003 | 611 (60.6) | HADS-A ≥ 11 |
| Depression diagnosis | 1003 | 245 (24.3) | HADS-D ≥ 11 |
| ***Psychological wellbeing*** | | | |
| Body Dysmorphic Disorder diagnosis | 1005 | 388 (38.5) | BICI score ≥ 72 |
